# Effect of water salinity on total protein and electrophoretic pattern of serum proteins of grass carp, *Ctenopharyngodon idella*

**Published:** 2014

**Authors:** Rahim Peyghan, Gholam Hosain Khadjeh, Ala Enayati

**Affiliations:** Department of Clinical Sciences, Faculty of Veterinary Medicine, University of Shahid Chamran of Ahvaz, Ahvaz, Iran.

**Keywords:** Electrophoresis, Grass carp, Salinity, Serum protein

## Abstract

In this study the effects of water salinity on serum total protein and its components in grass carp were investigated. The aim of this study was to determine the effect of salinity tolerance of fish on total serum protein level and its components as an indicator of liver and kidney activity. One hundred and twenty grass carp were divided into four groups, randomly. The first three groups were reared in concentration of 4, 8 and 12 g L^-1 ^of salt solution, respectively, and the fourth group was reared in freshwater and served as control. After 3 weeks, blood samples were collected and after harvesting the blood serum, serum total protein and protein components were measured with Biuret and electrophoresis methods, respectively. Results showed that mean value of serum total protein in the control and three salinities groups were 2.75, 3.28, 2.90 and 3.13 g dL^-1^, respectively. Five fractions of serum protein were electrophoretically observed as: albumin (Alb), alpha-1 globulin (α1-glu), alpha-2 globulin (α2-glu), beta globulin (β-glu) and gamma globulin (γ-glu). There were not any significant differences between the average mean of serum total protein of experimental and control groups (*p* > 0.05). However, Alb, α1-glu and β-glu levels in the experimental groups were significantly higher than in the control group (*p* < 0.05). The average of α2-glu and γ-glu revealed no significant difference between the experimental groups (*p* > 0.05). In conclusion, our results showed that increasing water salinity could have a significant effect on Alb, α1-glu and β-glu levels but not on total serum protein in grass carp.

## Introduction

Carp and most other bony fishes are able to regulate the ionic concentration of the internal body fluids within narrow ranges. Water salinity stress is one of the most common stresses in freshwater fishes that occasionally occurs and, if prolonged, can reduce production efficiency or can lead to death. In many studies, after the exposure of a freshwater fish to high water salinities (salinity stress), the fish was able to change the internal osmolarity of the plasma, allowing an influx of water.^1^^-^^4^

In some parts of Khuzestan province, water salinity of fish ponds may be higher than usual in some months of the year. Therefore, grass carp as a freshwater fish may be exposed to salinities higher than normal range. 

The importance of blood serum proteins and the finding that their values usually change in different physiological and pathological condition and also species variations necessitate determining the amount of total serum protein and its components.^5^^-^^7^

Serum proteins are the most important factors in blood and their clinical significance has been considered to be more in human and other mammals than the fish. Reportedly, Lepkovsky was the first to study fish blood serum proteins.^8^ Thereafter, other researchers presented more information about the differences and similarities in blood serum proteins in various fish species.^5^^,^^9^^-^^13^ Among different methods of fractionation of serum proteins, electrophoresis technique is one of the most widely used in different bases (Cellulose acetate, agarose gel, polyacrylamide gel) in this study. Since few studies on the serum proteins of the grass carp exist, therefore, it was necessary to study the normal level of blood proteins in this fish. This research was also done to determine the effect of increased salinity on total serum protein level and its components in grass carp.

## Materials and Methods

**Experimental groups. **One hundred and twenty grass carps were divided in to four groups randomly. The first three groups were reared in concentration of 4, 8 and 12 g L^-1^of salt solution, and the fourth group was reared in freshwater and served as control. The gradual increase of salinity in experimental groups was done within a period of three to four days.

**Sampling and measurements. **After three weeks, the fish were anesthetized and blood samples were collected from all fishes. For blood collecting, bleeding was done from the caudal peduncle vein. Blood samples were immediately transferred to sterile tubes and the serum was separated by centrifugation (1000 rpm for 10 min). Serum total protein was measured by Biuret method^14^ using biochemistry kit by spectrophotometer (Model M70; Bausch & Lomb Pharma NV, Brussel, Belgium) at 540 nm and serum protein components were fractionated by electrophoresis method, using cellulose acetate gel environment including Cellogel/Myl (Malta Chemetron Co., Milan Italy) and Triss Hipurate buffer (Malta Chemetron Co., Milan, Italy) with pH of 8.8, the electrophoresis tank (Akhtaryan Co., Tehran Iran) and power Supply (Paia Pajohsh Pars Medical Engineering Co., Tehran, Iran)**.** Electrophoresis time was 35 min and the voltage of 125 V was used. Hematoxylin and Eosin were used and staining lasted for 6 min. The value of protein fractions was determined by scanner and Photo-EP software. Photo-EP densitometry software (Hooshmand Fanavar Tehran Co., Tehran, Iran).

**Statistical analysis. **Mean values of each parameter were measured and compared in all groups by one way Analysis of Variance. The data were analyzed by SPSS software (Version 16; SPSS Inc., Chicago, USA) and considered significant at a level of *p* < 0.05. 

## Results

**Fig. 1:**
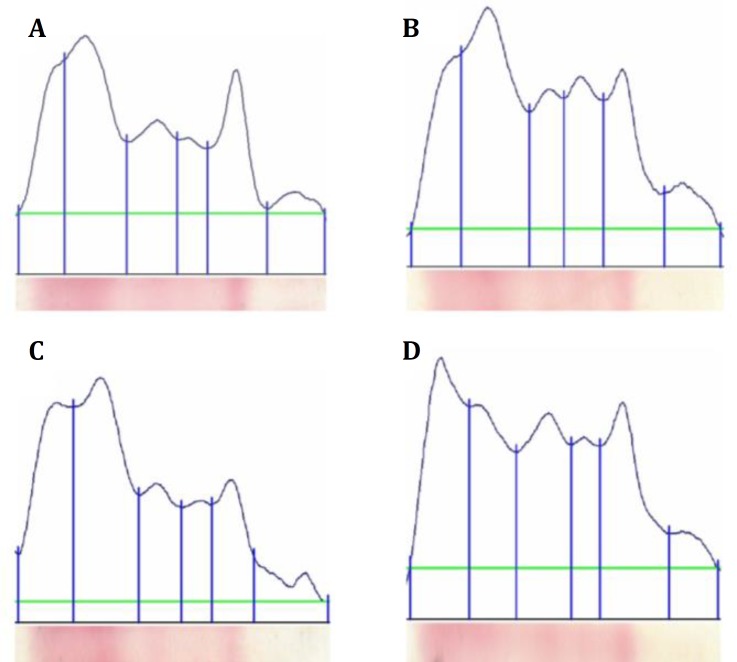
Electrophoretogram of serum protein of grass carp (*Ctenophayngodon idella)* in control group (A) and the experimental groups (B: 4 g L^-1^, C: 8 g L^-1 ^and D: 12 g L^-1 ^salinity). From left to right the sections comprise: pre-albumin, albumin, alpha-1, alpha-2, beta and gamma globulins

The results showed that, the average of serum total protein in the control and three salinity groups were 2.75, 3.28, 2.90 and 3.13 g L^-1^, respectively. In electrophoresis, serum proteins were fractionated in five fractions, including albumin (Alb), alpha-1 globulin (α1-glu), alpha-2 globulin (α2-glu), beta globulin (β-glu) and gamma globulin (γ-glu). Images of the blood serum protein or electrophoretogram patterns are shown in Figure 1. 

Our results showed that no significant difference was observed in the average of total serum protein in all groups, although Alb level in the 4 g L^-1 ^salinity group was significantly higher than in the control group (*p* < 0.05). In the 12 g L^-1 ^salinity group, Alb levels were decreased significantly in comparison with the 4 g L^-1 ^group (*p* < 0.05) and α1-glu levels in the 4, 8 and 12 g L^-1 ^salinity groups were decreased significantly in comparison with the control group (*p* < 0.05). Beta globulin levels in 8 and 12 g L^-1 ^salinity groups were decreased significantly in comparison with 4 g L^-1 ^and the control group (*p* < 0.05). The average of α2-glu and γ-glu did not show significant difference among groups (*p* > 0.05), (Table 1).

**Table 1:** The average (Mean ± SD) of serum total protein and protein fractions of grass carp in experimental groups and the control group

**Groups**	**تعداد ضربان قلب** **Albumin** **(g dL** ^-1^ **)**	**Alpha globulin-1** **RR** **(g dL** ^-1^ **)**	**Alpha2AAA** **Alpha globulin-2** **(g dL** ^-1^ **)**	**فاصل** **Beta globulin** **(g dL** ^-1^ **)**	**فاصله** **Gamma globulin** **(g dL** ^-1^ **)**
**گروه شاهد** **Control**	1.23 ±76328/3 0.35^a^	0.56 ±62068/2 0.10^a^	ᵅ 1136/0 ±0.26000.26 ± 03303/00.05	5612/0 ± 0.53 ± 10529/00.11^a^	6860/0 ±0.08 ± 12527/0 0.06
**Salinity 4 (g L** ^-1^ **)** **گروه4گر م**	0680/10 ± 2.06 ± 62955/4 0.27^b^	9514/6 ±0.380.38 ± 0.10^b^	ᵅ 1373/0 ± 0.24 ± 03169/00.18	5242/0 ± 0.54 ± 12176/00.05^a^	6681/0 ± 0.10 ± 10929/0 0.07
**گروه 8گرم** **Salinity 8 (g L** ^-1^ **)**	1765/12 ± 1.71 ± 43129/5 0.22^a^^b^	6507/5 ± 0.42 ± 90851/10.07^b^	ᵅ 1339/0 ± 0.27 ± 03102/00.04	4745/00.37± ±10966/ 0 0.04^b^	6233/0 ± 0.10 ± 11768/0 0.05
**گروه12گرم** **Salinity 12 (g L** ^-1^ **)**	1.377958/05768/4 ± 0.56^a^	6575/9 ± 0.37 ± 44013/40.070.07^b^	ᵅ 1242/0 0.25 ± 4333/ 0±0.05	6492/0 ± 0.38 ± 13296/00.08^b^	7971/0 ± 0.11 ± 15267/00.06

a, b Different letters in each column indicate significant difference (*p *< 0.05).

## Discussion

In this study the average of serum total protein and protein fractions were compared in different water salinities (experimental groups) and control groups. According to our results, no difference was observed in the average of total serum protein among all groups. Changes in concentration of serum total protein, in comparison to basic range, may be used as a clinical indicator in assessment of the health, stress status and body condition in aquatic species.^15^

The results of serum protein electrophoresis of grass carp led to the separation of five protein fractions, including: Alb, α1-glu, α2-glu, β-glu and γ-glu. In some cases six protein fractions were observed, including: pre-albumin, Alb, α1, α2, β and γ globulins (Fig. 1). Serum proteins play an important role in transport of different substances, defense of the organism against pathological agents, osmotic regulation, and some other functions.^16^ The effect of salinity on protein synthesis has not yet been studied. Salinity may have an effect on serum electrolytes and on liver metabolism. Rates of serum protein synthesis vary between tissues. The major tissues are liver, gill, gastrointestinal tract, kidney and white muscles. Liver has a central position in the synthesis and export of many proteins. Many factors that affect the liver function may have the potential to modify rates of protein synthesis and to differentially affect responses in different tissues.^17^

Among the fractionated proteins, Alb has been the fastest moving in the electric field, having a maximum value, while γ-glu is the lowest. Das studied blood biochemical parameters of three Indian carp species (*Catla Catla, Cirrhina *and* Labeo rohita*) including total protein and plasma protein.^18^ He used electrophoresis method for the fractionation and six fractions were observed as pre-albumin, Alb, α1, α2, β and γ globulin. In his study, there were significant differences among the values of α1, α2, β and γ globulins in the three species. Average of total protein and Alb in blood plasma of common carp, *Cyprinus carpio,* are reported by Nakagava *et al*.^19^ In this study, the total protein and Alb were 2.62 and 1.17 g dL^-1^, respectively, and six fractions were separated electro-phoretically, using cellulose acetate electrophoresis method.^19^ Manera and Britti separated the maximum six and minimum four fractions of the protein in rainbow trout blood serum using cellulose acetate electrophoresis method,^20^ Rehulka investigated ratio of total protein, Alb and Alb to globulin of *Cyprinus carpio*. Six protein fractions were separated by electrophoresis in blood serum, including: Alb, β-glu (three subsections) and γ-glu (with two sub-section).^8^^,^^21^ In another experiment ,Pike-Perch (*Sander lucioperca*) were held in two different salinities at concentrations of 9 and 12 g L^-1^ for 11 days and the results indicated that no significant difference was observed in blood proteins between treatments.^21^^,^^22^ According to the results of the present study, the average of serum total protein and also the average of α2-glu and γ-glu did not show significant difference between groups (*p* > 0.05). 

In our study, Alb level in the 4 g L^-1 ^salinity group was significantly higher than in the control group (*p *< 0.05) but in 12 g L^-1^ salinity group, Alb levels were decreased significantly in comparison with 4 g L^-1^ group (*p *< 0.05). Albumin main function is reported as the regulation of colloidal osmotic pressure of the blood and transport of some exogenous components such as drugs and endogenous chemicals (i.e. fatty acids, hormones, and bilirubin).^22^^-^^24^ Concentration of albumin-like proteins in fish plasma of teleosts was reported from 10.00 to 50.00%, while in terrestrial vertebrates Alb accounts for more than 50.00% of the total serum proteins concentration.^25^ Albumin-like fractions are identified in some other teleost fish species by electrophoretic method.^26^

Alpha globulin-1 level, in 4, 8, 12 g L^-1 ^salinity groups was decreased significantly in comparison with the control group (*p *< 0.05). Beta globulin levels in the 8, 12 g L^-1 ^salinity groups were decreased significantly in comparison with 4 g L^-1 ^and the control group (*p *< 0.05). Farghaly *et al*. studied the effect of temperature and salinity changes on blood parameters of *Tilapia* *zilli*. They found that the alpha and beta globulins, and also the blood coagulation time were decreased as salinity increased ^27^. Imanpoor *et al*. investigated the effects of different salinities (0, 6 and 12‰) and temperatures (23, 27 and 31 ˚C) on the food consumption, growth rate and blood biochemistry, and hematocrit value of Goldfish.^28^ Their results suggested that the plasma total protein levels were decreased with the increase in salinity, while they were independent for temperature. 

Our results showed that an increase in water salinity can have a significant impact on electrophoretic pattern of serum proteins in grass carp, *Ctenophayngodon idella*. 

The structural reorganizations of proteins in condition of increased salinity can account for the increase in protein level.^29^ In the carp, the structural reorganization of albumins was described during trans capillary exchange under adaptation of the fish to increased salinity (8, 10, 11 and 20‰), consisting of dissociation of oligomeric protein into its subunit components in interstitial fluid.^30^ As the capillary walls of fish are non-permeable to proteins of blood plasma,^29^^-^^32^ it is probable that the increased permeability of fish capillary walls may account for the changes in concentration and the level of blood of the studied fish.
